# High-Frequency Percussive Ventilation in Cystic Fibrosis Patients With Acute Respiratory Failure: A Case Series

**DOI:** 10.7759/cureus.16087

**Published:** 2021-07-01

**Authors:** Badr Jandali, Joel D Mermis, Michael S Crosser

**Affiliations:** 1 Pulmonary and Critical Care Medicine, The University of Kansas Health System, Kansas City, USA

**Keywords:** cystic fibrosis, mechanical ventilation, high frequency percussive ventilation, acute respiratory failure, airway clearance

## Abstract

Acute respiratory failure in cystic fibrosis carries a high risk of mortality. The optimal mode of mechanical ventilation (MV) in this population is not well established. In this case series, we identified patients with cystic fibrosis who were ventilated with high-frequency percussive ventilation (HFPV) at our institution and describe their characteristics and outcomes. The use of high-frequency percussive ventilation has been sparsely described in the literature. This case series could serve as hypothesis-generating for future research.

## Introduction

Acute respiratory failure in cystic fibrosis (CF) carries a significant risk of morbidity and mortality [1]. The short and long-term outcomes are significantly worse when mechanical ventilation (MV) is needed. The optimal ventilation mode for this patient population is unknown. The benefits of chest physiotherapy and airway clearance in CF are well known [2]. This becomes of utmost importance during respiratory failure. However, once on MV, the conventional means of airway clearance are generally of limited effectiveness as patients are generally sedated and unable to complete the necessary maneuvers to facilitate effective airway clearance. Thus, on MV, accumulation of inspissated infected sputum can lead to further compromise and worsening respiratory failure. High-frequency percussive ventilation (HFPV) is a time-cycled, pressure-limited mode of percussive ventilation that delivers subphysiologic tidal volumes at rates that can exceed 500 breaths per minute. In other pulmonary diseases with airway debris, HFPV is employed as the percussive airflow facilitates secretion mobilization and clearance after endotracheal intubation takes place [3]. While this approach has not been studied in CF, the use of HFPV in thermal inhalation injuries has consistently shown less incidence of ventilator-associated pneumonia (VAP), improved oxygenation and ventilation, and possibly improved mortality [3-5].

We reviewed the available literature and could only find a single case report of using HFPV in a cystic fibrosis pediatric patient [6]. Given poor outcomes on conventional MV and mechanistic plausibility supporting HFPV, we have been using this mode of ventilation at our institution with our adult CF population. We aim to describe our experience with this case series.

## Materials and methods

The institutional review board approval for data collection was obtained before any data gathering. The medical record Epic (Epic Systems, Verona, USA) was queried using HERON (Healthcare Enterprise Repository for Ontological Narration) software (University of Kansas Medical Center, Kansas City, USA) with appropriate ICD-9/ICD-10 codes to identify patients who had cystic fibrosis and required mechanical ventilation using high-frequency percussive ventilation. A list of 12 patient encounters was identified. This was cross-referenced and confirmed with our departmental CF registry. Patients' charts were individually checked to confirm cystic fibrosis diagnosis (defined by sweat chloride > 60 mmol/L or two known CF-causing mutations) and identify those who were ventilated using HFPV. One patient was excluded due to lung transplant status and one patient had two encounters that are reported separately. The identified patients were hospitalized between April 2010 to May 2019 and data was collected in September 2020. Baseline forced expiratory volume in the first second (FEV1) was defined as the most recent FEV1 measured at an office visit prior to admission in a stable condition and post-hospital FEV1 was defined as the FEV1 measured at the first office visit post-hospitalization.

## Results

Eleven patient encounters (four females) with CF and acute respiratory failure treated with HFPV were analyzed. Six patients survived to discharge and clinic follow-up. The median age was 30 (20-40) years. The median hospital length of stay (LOS) was 28 (15-37) days while the ICU median LOS was 9 (4-24) days. The median time spent on total MV was 4 (3-12) days and on HFPV 4 (2-11) days. The median baseline FEV1 prior to admission was 1.04L (0.73-1.62 L), 29% (19-45%) of predicted (Table 1).

**Table 1:** Patients' characteristics CF: cystic fibrosis; MV: mechanical ventilation; HFPV: high-frequency percussive ventilation; DIOS: distal intestinal obstructive syndrome; P A/C: pressure assist control; PRVC: pressure regulated volume assist; SIMV: synchronized intermittent mandatory ventilation

Patient	Age(Y)	sex	Hospital stay (days)	ICU stay (days)	Admission diagnosis	Mutation	Respiratory failure cause	Days on MV	Days on HFPV	Other modes of ventilation	Vasopressor use	Acute renal failure	Survived
01	23	M	52	52	Respiratory failure	Homozygous del F508	Pneumonia	53	11	P A/C	yes	yes	No
02	65	M	8	4	CF pulmonary exacerbation	delF508 and P205S	Pneumonia	4	4	P A/C	yes	yes	No
03	22	F	12	4	Sepsis	Homozygous del F508	Pneumonia	3	2	P A/C	no	no	Yes
04	30	M	15	3	CF pulmonary exacerbation	del F508/V753M	Pneumonia	2	2	PRVC	no	no	Yes
05	30	M	30	13	DIOS	del F508/V753M	Pneumonia	12	12	PRVC	yes	yes	No
06	22	M	39	5	CF pulmonary exacerbation	Homozygous del F508	Pneumonia	4	3	SIMV	no	no	Yes
07	30	F	30	24	DIOS/hemoptysis	N/A	Pneumonia	4	4	P A/C	yes	yes	No
08	46	M	28	17	CF pulmonary exacerbation	N/A	Pneumonia	9	5	P A/C	yes	yes	Yes
09	40	F	23	5	CF pulmonary exacerbation	N/A	Pneumonia	3	2	P A/C	yes	yes	No
10	22	M	37	26	CF pulmonary exacerbation	Homozygous del F508	Pneumonia	18	16	P A/C	no	no	Yes
11	22	F	27	9	CF pulmonary exacerbation	Homozygous del F508	Pneumonia	4	2	PRVC	no	no	yes

**Figure 1:**
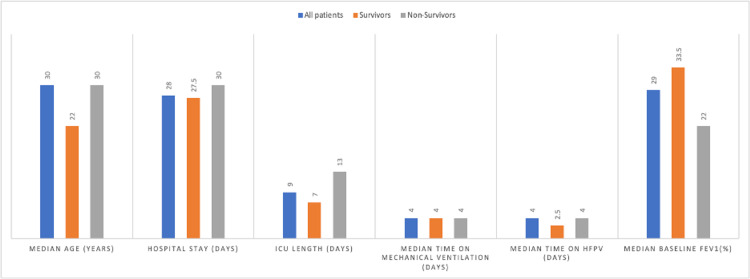
Comparison of survivors and non-survivors

The six patients who survived to hospital discharge had a median age of 22 (22-34) years. Their hospital median LOS was 27.5 (14.25-37.5) days and median ICU LOS was 7 (3.75-19.25) days. They spent a median of 4 (3.75-11.25) days on total MV. Of those, a median of 2.5 (2-7.75) days were spent on HFPV (Figure 1).

**Figure 2:**
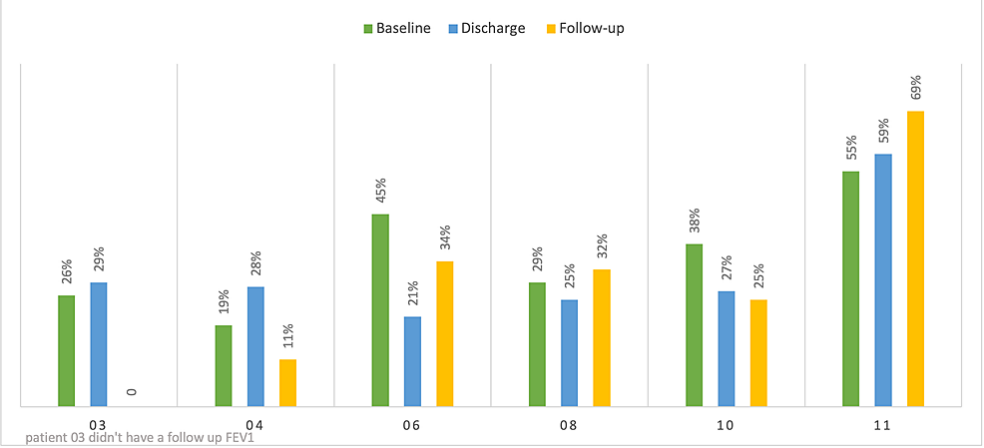
FEV1 trends of the patients who survived Patient 3 went to transplant shortly post-discharge and had no follow-up FEV1 on our records. FEV1: forced expiratory volume in the first second.

Their median prior-to-admission baseline FEV1 was 1.41 L (33.5% of predicted) with a median follow-up FEV1 of 1.33 L (32% of predicted) (Figure 2).

All the patients in this cohort had documented improved airway clearance by the ICU team after implementing HFPV without documented complications. However, this finding was limited to subjective observation and was not quantified by a standard method.

## Discussion

High-frequency percussive ventilation is a subtype of high-frequency ventilation. It is a time-cycled and pressure-limited ventilator that delivers small tidal volumes using inspiratory and expiratory oscillations. The ventilator is called Volumetric Diffusive Respiration Unit (VDR-4) and comes with a Phasitron (Percussionnaire, Sandpoint, USA). This is an inspiratory and expiratory valve that is driven by a high-pressure and high-frequency gas supply superimposed on conventional pressure-controlled cycles [7].

There are multiple mechanisms that contribute to gas exchange during HFPV. Aside from the direct bulk flow that is observed with conventional ventilation, HFPV creates an asymmetric velocity profile with a laminar flow pattern in which gas in the center of the airway advances inward and gas outside the center flows in a retrospect fashion [8]. This flow pattern along with the continuous pulsatory percussive waves, aid the lysis and mobilization of inspissated viscous secretions in cystic fibrosis patients [9]. 

While it is possible to use conventional mechanical ventilation with intermittent intrapulmonary percussive pressure, HFPV offers the potential advantage of continuous secretions mobilization and minute-to-minute airway clearance for CF patients. This is especially beneficial when cough reflexes are suppressed with sedation and when mucous production is at a peak with active infection.

In this case series, we report the outcomes of 10 cystic fibrosis patients (11 hospital encounters) in whom HFPV was used during invasive MV. While small, this CF cohort does appear to represent the adult CF ICU patient in terms of initial diagnosis, the severity of illness, and age. All of the patients were admitted with primary respiratory and CF-related diagnoses. All of the MV indications were directly related to primary respiratory failure. As expected, the median baseline FEV1 of 29% does suggest advanced CF lung disease and poor lung function at baseline.

The in-hospital mortality in this case series is five out of 11 patients, which is consistent with a recent national cross-sectional study where Siuba et al found a mortality rate of 44.5% for cystic fibrosis patients requiring MV [1]. The modes of ventilation were not defined in that study, but our numbers would at least suggest mortality outcomes consistent with the national trend. Respiratory therapy documentation and physician progress notes all noted significant improvement in airway clearance (secretion mobilization via endotracheal tube) after HFPV implementation, but this finding was not uniformly quantified by a standard method and could be prone to bias. However, it is consistent with the authors' experience and with non-CF trials on HFPV, especially in thermal inhalation injuries. In addition, the HFPV offers an added advantage of using lower peak airway pressures and decreases the risk of dynamic hyperinflation by using smaller tidal volumes. None of the patients in our cohort suffered from pneumothorax or ventilator-related complications.

## Conclusions

High-frequency percussive ventilation appears to be safe, has the potential to augment airway clearance, and is at least as effective as conventional ventilation for cystic fibrosis patients who require invasive MV. Further trials would be needed to demonstrate superiority versus the current standard of care.
